# A Deeper Microscopic Study of the Interaction between Gum Rosin Derivatives and a Mater-Bi Type Bioplastic

**DOI:** 10.3390/polym12010226

**Published:** 2020-01-16

**Authors:** Miguel Aldas, Emilio Rayón, Juan López-Martínez, Marina P. Arrieta

**Affiliations:** 1 Departamento de Ciencia de Alimentos y Biotecnología, Facultad de Ingeniería Química y Agroindustria, Escuela Politécnica Nacional (EPN), Quito 170517, Ecuador; 2 Instituto de Tecnología de Materiales, Universitat Politècnica de València (UPV), 03801 Alcoy-Alicante, Spain; jlopezm@mcm.upv.es; 3 Facultad de Ciencias Químicas, Universidad Complutense de Madrid (UCM), Avenida Complutense s/n, Ciudad Universitaria, 28040 Madrid, Spain

**Keywords:** biodegradable polymers, thermoplastic starch, gum rosin, gum rosin esters

## Abstract

The interaction between gum rosin and gum rosin derivatives with Mater-Bi type bioplastic, a biodegradable and compostable commercial bioplastic, were studied. Gum rosin and two pentaerythritol esters of gum rosin (Lurefor 125 resin and Unik Tack P100 resin) were assessed as sustainable compatibilizers for the components of Mater-Bi^®^ NF 866 polymeric matrix. To study the influence of each additive in the polymeric matrix, each gum rosin-based additive was compounded in 15 wt % by melt-extrusion and further injection molding process. Then, the mechanical properties were assessed, and the tensile properties and impact resistance were determined. Microscopic analyses were carried out by field emission scanning electron microscopy (FE-SEM), atomic force microscopy (AFM) and atomic force microscopy with nanomechanical assessment (AFM-QNM). The oxygen barrier and wettability properties were also assayed. The study revealed that the commercial thermoplastic starch is mainly composed of three phases: A polybutylene adipate-co-terephthalate (PBAT) phase, an amorphous phase of thermoplastic starch (TPSa), and a semi-crystalline phase of thermoplastic starch (TPSc). The poor miscibility among the components of the Mater-Bi type bioplastic was confirmed. Finally, the formulations with the gum rosin and its derivatives showed an improvement of the miscibility and the solubility of the components depending on the additive used.

## 1. Introduction

Despite the favorable attributes of petroleum-derived plastics, there is a concern about their high consumption, which turns into the accumulation of plastic waste in landfills and marine environments [1,2,3]. Therefore, to reduce their environmental impact, research has been promoted to find alternative more environmentally friendly plastics [1,4,5,6,7]. Nevertheless, the currently commercialized bioplastics still present some drawbacks with respect to traditional plastics, currently used in short term applications [7]. These alternative sustainable polymers must also have acceptable mechanical, physical, and morphological properties, to allow them to have similar characteristics to those of traditional plastics [1,3,8].

In this context, as sustainable alternatives, biopolymers have gained considerable interest for short term applications. Polymer blending is a very interesting approach to tune up the physical and mechanical properties of biopolymers since it is a common and relatively simple process already available at industrial level [7,9]. Among other bioplastics, a greater interest on thermoplastic starch (TPS) has been placed, because the starting material (starch) is an abundant and cheap material coming from renewable sources, while it is also biodegradable [10].

For industrial applications, starch is preferably used in its thermoplastic form, that is TPS, rather than in its native form, because the plasticized granules of native starch allow the formation of a continuous and viscous phase, which can be easily processed by traditional plastics processing technologies [8,11]. In fact, during the extrusion process the granular structure of native starch is disrupted, combined by the action of temperature and shear forces and, in the presence of a plasticizer, it forms a melted polymeric system, that is the so called thermoplastic starch (TPS) [12]. However, neat TPS has two disadvantages for industrial applications as packaging material: Its solubility in water and its low mechanical properties. Thus, to solve this inconvenience, in commercial TPS grades physical blends or copolymers from vinyl alcohol or aliphatic-aromatic polyesters or copolyesters are frequently prepared. Mineral fillers or natural fibers can also be added to improve their properties [13,14,15].

One of the most popular commercial TPS is Mater-Bi^®^, a family of modified biodegradable and compostable thermoplastic starches produced by Novamont [16]. Mater-Bi^®^ mainly consists of corn starch and various synthetic compounds [8,14,17,18], including natural plasticizers and hydrophilic substances biologically degradable from synthetic polymers [11,13]. Although the exact composition of Mater-Bi^®^ is not known, the literature refers to certain indications [15,17]. Depending on Mater-Bi^®^ material composition it presents different properties. Thus, it is possible to find: (i) Mater-Bi^®^ Y, composed starch and cellulose acetate blends, whose properties resemble those of polystyrene (PS); (ii) non-compostable Mater-Bi^®^ A, constituted by a strong complex between TPS and copolymers of polyvinyl alcohol (PVA); (iii) Mater-Bi^®^ V, having a TPS content greater than 85% and a high solubility in water [14]; (iv) Mater-Bi^®^ Z, having a poly(ε-caprolactone) (PCL) matrix; and (v) Mater-Bi^®^ N whose base polymeric matrix is polybutylene adipate-co-terephthalate (PBAT) [5,14].

In this work, Mater-Bi^®^ NF866 of class N has been employed. There is little scientific information available about it [14], but it is known that it is mostly based on TPS and PBAT blend [5,6]. It is also presumed that the composition of Mater-Bi^®^ N-type contains some compatibilizers since PBAT is a hydrophobic polymer and TPS has a hydrophilic character, which from the thermodynamic point of view makes these materials immiscible, leading to having very low interfacial adhesion and phase separation [19,20]. Borchani et al. (2015) determined that the composition of Mater-Bi ^®^ is 70%, 20%, and 10% of PBAT, starch, and additives, respectively [5]. In this work, this class of Mater-Bi has been selected since it is a material developed as an alternative to polyethylene-based plastics. It was designed to be suitable for blow extrusion process and to obtain thin biodegradable films used in the agricultural and packaging industries [21].

On the other hand, there is a growing tendency on the use of additives derived from natural resources to develop biobased polymeric systems to guarantee the green nature of the final polymeric material to be used as potential substitutes of some conventional synthetic polymers consumed in short term applications [3]. Many advantages of gum rosin and its derivatives such as their easy availability, low cost, and its renewable origin from non-food crops make them interest as sustainable additives for the next generation of food packaging or agricultural sustainable plastic materials [22]. In fact, pine resin derivatives have gained attention as toughening additives for plastics [22,23,24]. Moreover, considering that resin production is a defensive response of pines to external factors (e.g., insect or pathogen attack, mechanical wounding) which results in localized accumulation of resin [25], pine cleaning activities are required for good forest management practices to avoid fires [22]. Moreover, the use of non-food crops for biodegradable plastic applications is strongly recommended [26]. Thus, it is very interesting to revalorize pine resin and its derivatives as sustainable additives for the plastic industry, since they are not only biobased, but also come from non-food crops and therefore they will not compete with food supply [22].

In a previous work [8], to counteract the low interfacial adhesion between PBAT and starch in Mater-Bi^®^ NF866, different pine resin derivatives were used as Mater-Bi^®^ NF866 sustainable additives. As a result, several materials with different varieties of mechanical, thermal, and processability properties were obtained; depending on the type of pine resin employed thanks to the plasticizing, solubilizing, and/or compatibilizing effect produced due to the presence of gum rosin derivatives. Moreover, it was observed that the amount of 15 wt % was enough to tune the mechanical properties of Mater-Bi^®^ bioplastic. Nevertheless, the behavior in the diversity of the properties was not covered from a deeper microscopic point of view, allowing for information of the miscibility of the components of the polymeric system and its influence on the mechanical and permeability performance. Therefore, in the present study, with the main propose to delve into the analysis of the interaction between gum rosin derivatives and the commercial thermoplastic starch Mater-Bi^®^ N-type, we have studied the microscopic aspects of the interaction among all the components in the polymeric system to get insights on the influence of their miscibility, not only on the mechanical properties, but also on the permeation performance. First, the materials were characterized by mechanical properties. Then, several microscopy analysis techniques were employed (field emission scanning electron microscopy, FE-SEM; atomic force microscopy, AFM; and atomic force microscopy with nanomechanical assessment, AFM-QNM) with the main objective to study the miscibility of the components of Mater-Bi^®^ NF 866 bioplastic as well as their microscopic interaction as a consequence of the presence of non-modified gum rosin. Finally, since these materials are intended for the agricultural and/or food packaging sector, surface wettability and oxygen barrier performance have been evaluated and related with the miscibility of Mater-Bi^®^ NF866 bioplastic components as a consequence of the use of non-modified gum rosin or pentaerythritol esters of gum rosin.

## 2. Materials and Methods

### 2.1. Materials

Mater-Bi type bioplastic (labeled as MB), based on starch and aliphatic-aromatic polyesters (PBAT and PCL), under the trade name Mater-Bi^®^ NF 866 was supplied by Novamont SPA (Novara, Italy). The MB has a melting temperature in the range of 110 to 120 °C and a melt index of 3 g/10 min at 150 °C with a load of 5 kg [17,18].

The additives blended with the MB biopolymeric matrix were gum rosin and two pentaerythritol esters of gum rosin. The gum rosin or colophony (labeled as GR) was supplied by Sigma-Aldrich (Mostoles, Spain). The esters were: (i) Unik Tack P100 resin kindly supplied by United Resins (Figueira da Foz, Portugal), with a softening point of 90 °C, an acid number of 15 and Gardner color of 4 (labeled as UT); and (ii) Lurefor 125 resin kindly supplied by LureSA (Segovia, Spain), with a softening point of 125 °C, an acid number of 11.9 and Gardner color of 7 (labeled as LF). As described, LF and UT differ from each other in the acid number, which denotes that the number of functionalized groups in the modified structure is greater in LF than in UT [27,28,29].

### 2.2. Samples Preparation

All materials were dried at 50 °C for 24 h in an air circulation oven previous to being processed. Then, MB was melt blended with 15 wt % of gum rosin derivatives in a twin-screw extruder (Dupra S.L, Castalla, Spain), using a temperature profile of 160, 150, 140, 100 °C (from die to hopper) at 50 rpm. The blends were further milled into pellets to be processed by injection-molding (Sprinter-11, Erinca S.L., Barcelona, Spain). The injection temperature profile was established based on previous work [8]. That is, from die to hopper, the temperatures for the MB matrix were 165, 160, 160 °C; for the blend of MB and 15 wt % of gum rosin (label as MB-GR), temperatures were 117, 117, 105 °C. For the formulations with 15 wt % of gum rosin derivatives (LF and UT), labelled as MB-LF and MB-UT, respectively, the profile was 145, 140, 135 °C for MB-LF and 120, 115, 105 °C for MB-UT. The samples obtained were standard rectangular specimens (80 × 10 × 4 mm^3^).

### 2.3. Sample Characterization

#### 2.3.1. Tensile Test

Mechanical properties were evaluated by means of tensile test measurements which were performed according to standard test methods ISO 527 [30]. The tests were performed in a universal test machine Ibertest ELIB-50-W (Madrid, Spain) with a load cell of 5 kN and a crosshead rate of 10 mm/min. Charpy’s impact resistance was also assessed in a Metrotec impact equipment (San Sebastian, Spain), using a 1 J pendulum and A-type notched specimens under the ISO 179 [31]. For each test five specimens were characterized, and the mean and standard deviation of the values were reported. The significance in the mechanical data differences was statistically analyzed using the one-way analysis of variance (ANOVA), by means of OriginPro 8 software, at 95% confidence level according to Tukey’s test for the significant differences among formulations.

#### 2.3.2. Dynamic Thermo-Mechanical Analysis (DTMA)

Dynamic thermo-mechanical analysis (DTMA) of the samples was performed in an oscillatory rheometer AR G2 from TA Instruments (New Castle, England), in torsion mode. The rheometer was equipped with a special clamp system for solid samples. The test samples were rectangular samples sized 40 × 10 × 4 mm^3^ and were fabricated by injection-molding from the previously obtained pellets of each formulation. The temperature range of the test was set from −50 to 110 °C and the heating rate was set on 2 °C/min. The frequency was 1 Hz and the maximum deformation 0.1%. A liquid nitrogen atmosphere was employed during the tests. The glass transition temperatures (T_g_) were determined at the maximum peaks of the tangent of the loss factor (tan δ).

#### 2.3.3. Microscopic Characterization

The microstructure of the different formulations was observed by field emission scanning electron microscopy (FE-SEM), using a Zeiss Ultra 55 microscope at 1 kV. Samples of 1 µm thick were obtained by the environmental ultramicrotomy of the central part of the previously injected specimens. Through this technique, it was possible to obtain sufficiently smooth and flat surfaces for observation by FE-SEM and atomic force microscopy (AFM), while it was possible to appropriately reveal the microstructure of the material.

The atomic force microscopy with nanomechanical assessment (AFM-QNM) was performed in an AFM model Nanoscope II from Veeco National Instrument (Santa Barbara, CA, USA) working in peak force tapping mode by the quantitative nanomechanical measurement. QNM method based on literature [32,33] has been used. With the AFM-QNM analysis of the topography map, deformation, stiffness, and adhesion of each area was obtained [34]. An antimony (n) doped Si cantilever of 5 N/m and with a Poisson coefficient of 0.48 was used. The radius of the tip was determined at 20 nm by a calibration carried out in a standard sample of polystyrene at 5 nm, the same depth that the contact with the sample was made during the test. The calibration of the area function was done before and after the test, to certify that there was no wear during the entire test procedure. Derjagin, Muller, Toropov model (DMT) was employed to calculate the elastic modulus. This model takes into account the contact adhesive component [32].

#### 2.3.4. Oxygen Transmission Rate (OTR)

The oxygen transmission rate (OTR) was measured with a Systech Instruments 8500 oxygen permeation analyzer (Metrotec S.A, San Sebastián, Spain) operating at room temperature and 2.5 atm. To prepare the appropriate samples for OTR measurements, masterbatch pellets were processed into 14 cm diameter film discs by using a hot press (Mini C 3850, Caver, Inc., Wabash, IN, USA) with the following pressure cycle: At atmospheric pressure for 5 min, 3 MPa for 1 min, 5 MPa for 1 min, and 10 MPa for 3 min. The films were then quenched to room temperature at atmospheric pressure. Their average thickness was around 450 μm. The film disks films were compressed between the upper and lower diffusion chamber. Pure oxygen (99.9% purity) was introduced into the upper half of the sample chamber while nitrogen was injected into the lower half. The oxygen volumetric flow rate per unit area of the film and per time (OTR, cm^3^ × mm m^−2^ × day^−1^) was continuously monitored until a steady state was reached. Measurements were expressed as oxygen transmission rate per film thickness (OTR·*e*).

#### 2.3.5. Static Contact Angle Measurements

Surface wettability was studied through static water contact angle (WCA) measurements by using a standard goniometer (EasyDrop-FM140, KRÜSS GmbH, Hamburg, Germany) equipped with a camera and Drop Shape Analysis SW21; DSA1 software. Ten contact angle measurements were taken in random positions, putting drops of ~2 μL distiller water onto the surface of the samples with the aid of a syringe. The average values were calculated and reported.

## 3. Results and Discussion

### 3.1. Tensile Test

**Figure 1:**
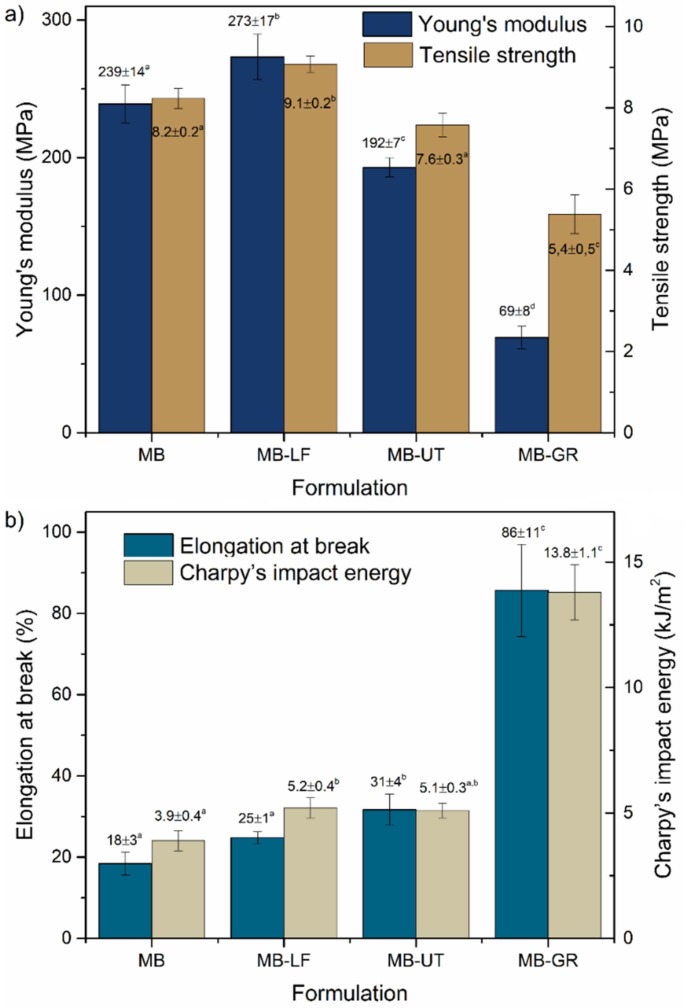
Mechanical properties of the Mater-Bi^®^ NF 866, Mater-Bi^®^ NF 866 with Luerefor 125 resin, Mater-Bi^®^ NF 866 with Unik Tack P100 resin, and Mater-Bi^®^ NF 866 with gum rosin: Young’s modulus and tensile strength (**a**) and elongation at break and Charpy’s impact energy (**b**). ^a–d^ Different letters within the same property indicate statistically significant differences between formulations (*p* < 0.05).

The mechanical values for the studied formulations were obtained previously [8], and are reported in this study for comparison. Figure 1 shows a comparative graphic of the mechanical properties of the studied formulations.

Both properties follow the same tendency. That is, for MB, MB-LF, and MB-UT, both properties have higher values than MB-GR. The incorporation of UT and LF does not highly affect the modulus, nor the tensile resistance. However, LF resin contributes to having more cohesion in the material, and therefore, higher resistance and modulus values. On the contrary, a 15 wt % of GR reduces the modulus of the material in approximately 70%, and the tensile strength in about 35% compared to the MB. This is indicative of the plasticizing and compatibilizing effect of the resins. From one side UT and LF act as compatibilizers for the MB components and from the other side, GR acts as plasticizer of the components. All formulations show significant differences in the values of the properties (*p* < 0.05).

Regarding the elongations at break and the Charpy’s impact energy shown in Figure 1b, once again both properties show the same trend. Both properties increase regardless of the type pine resin suggesting a solubilizing, compatibilizing, and plasticizing effect, particularly in the case of MB-GR. This formulation exhibits a great increase of the elongations at break as well as the impact resistance as a result of the already observed plasticizing effect. In this formulation, the impact resistance increases 350%, meanwhile the elongation at break increases more than 470% compared to the matrix, increasing the ductility to the matrix and therefore, showing a higher solubility of GR in the MB. The other formulations (MB-LF and MB-UT) show similar properties to the MB, with just slight significant differences (*p* < 0.05), corroborating the compatibilizing effect of the UT and LF, already reported in literature [8].

In all the studied formulations, an increase in the cohesion of the components of the material is observed, although the properties are different depending on the type of additive. In general, the mechanical properties of the studied formulations show an improvement in the final performance of the blends, compared to the matrix. Nevertheless, it is not possible to assess the specific behavior or interaction of each resin with the components of the MB matrix, by the analysis of the mechanical properties. For that reason, a deeper study of the effect of the resins with the components of the matrix, by means of microscopic technique, is presented in the following sections.

### 3.2. Dynamic Thermo-Mechanical Analysis (DTMA) Characterization

**Figure 2:**
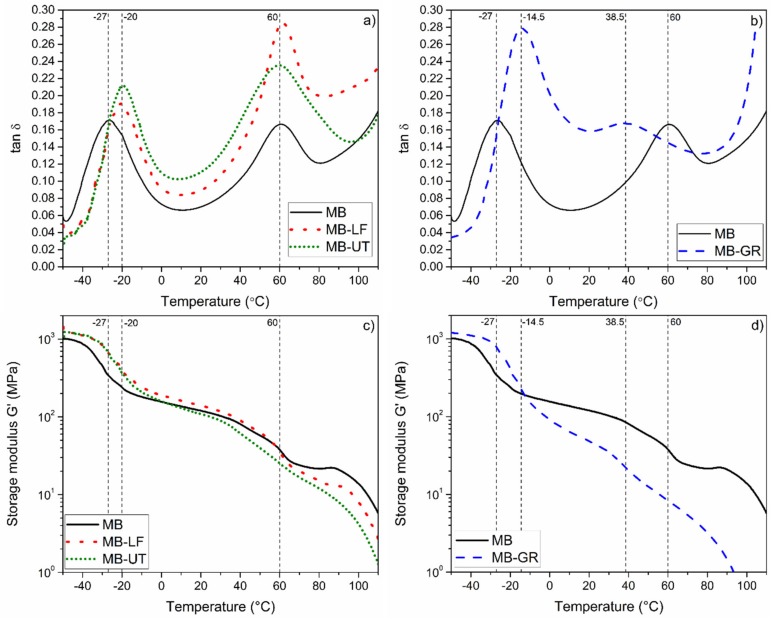
Dynamic thermo-mechanical analysis (DTMA) curves of the loss factor (**a**,**b**) and the storage modulus (**c**,**d**) of Mater-Bi^®^ NF 866, Mater-Bi^®^ NF 866 with Luerefor 125 resin, Mater-Bi^®^ NF 866 with Unik Tack P100 resin, and Mater-Bi^®^ NF 866 with gum rosin. The main transition temperatures are specified.

DTMA was used to study the effects of gum rosin derivatives on the compatibility of MB material and were reported in a previous work [8]. The evolution with the temperature of the loss factor (tan δ) (that is the gap between the loss modulus (G”) and the storage modulus (G’), represented by the tangent of the gap (tan δ)), is plotted in Figure 2a,b. In the case of the curve corresponding to MB, it is possible to see two maximum peaks at −27 and 60 °C. The first one is associated to the glass transition temperature (T_g1_) of the soft segment (aliphatic) of the PBAT portion and the secondary glass transition of plasticizer-rich domains in the MB. The second one (T_g2_) is a conjugated peak linked to both, the hard segment (aromatics structures of the PBAT) as well as a primary glass transition of starch-rich phase of the MB, respectively [8,35,36]. This behavior suggests low miscibility among the components of the Mater-Bi type bioplastic. From DTMA curves of MB-LF and MB-UT formulations, reported in Figure 6a, it is possible to confirm that the incorporation of the gum rosin derivatives allow a shift of T_g1_ peak, from −27 to −20 °C, meanwhile the T_g2_ peak remains constant. On the other hand, in the case of MB-GR formulation (Figure 6b) both peaks are modified, T_g1_ from −27 to −14.5 °C and T_g2_ from 60 to 38.5 °C. This last peak is also transformed from a sharp peak to a shoulder peak. This behavior is consistent with the plasticizing and compatibilizing effects of the GR [8]. These variations in the shape of the curves and the shifts of the glass transition temperatures suggest that the gum rosin derivatives help to increase the miscibility and the solubility among the components of the MB type bioplastic. Further, the compatibility conferred by the GR to MB-GR formulation is greater than the compatibility of MB-UT or MB-LF formulation, as has been recently discussed by Zhang et al. (2019) [36].

Figure 2c,d show the evolution with the temperature of the storage modulus (G’), for the studied materials. It can be seen that all the storage modulus of the formulations decrease when the temperature increases. Moreover, the main drops of the modulus of all the formulations take place in the same temperatures discussed above, which are attributed to the PBAT and starch phases of the MB, respectively. For temperatures below 0 °C, MB-LF, MB-UT, and MB-GR have higher G’ values than the MB, which is a consequence of the compatibility and interaction between the gum rosin derivatives and the components of the Mater-Bi type bioplastic. In contrast, for temperatures between 0 and 60 °C (T_g2_), the storage modulus of the UT and GR added materials decreased compared to the MB. Meanwhile, for MB-LF the modulus remains slightly higher than the one for MB. This behavior is in good agreement with the results obtained in the mechanical characterization, and it is explained by the plasticizing and compatibilizing effect of the studied resins; the solubilizing and compatibilizing effect of the GR and UT; and the cohesive effect of LF.

### 3.3. Field Emission Scanning Electron Microscopy (FE-SEM) Microstructural Characterization

**Figure 3:**
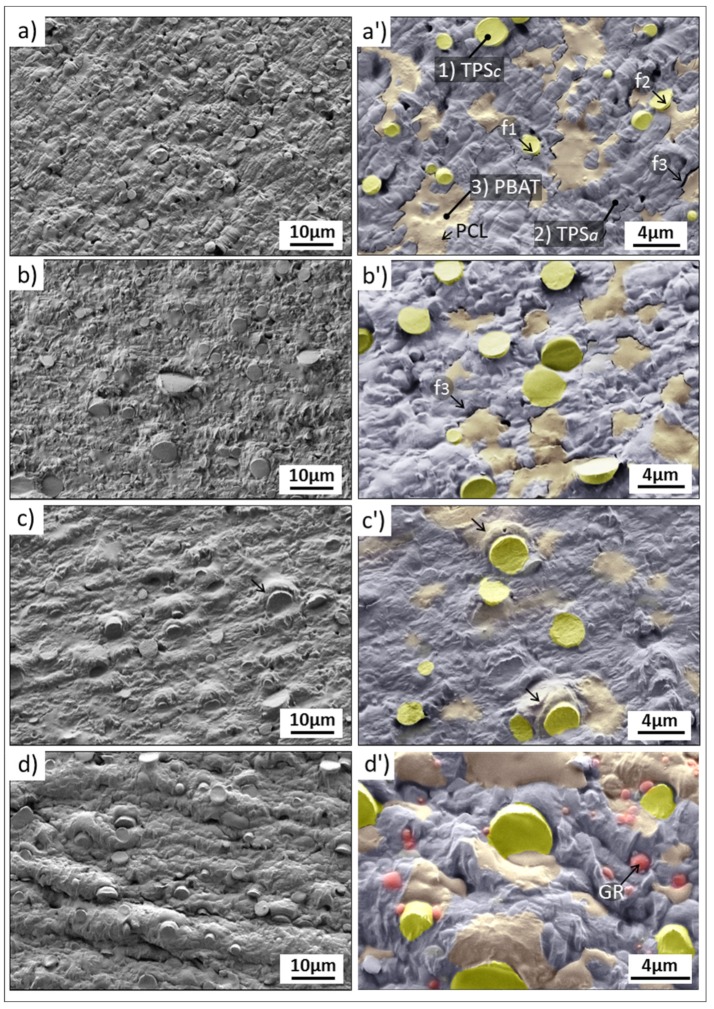
Colored field emission scanning electron microscopy (FESEM) images acquired at two magnifications on: Mater-Bi^®^ NF 866 (**a**,**a’**), Mater-Bi^®^ NF 866 with Luerefor 125 resin (**b**,**b’**), Mater-Bi^®^ NF 866 with Unik Tack P100 resin (**c**,**c’**), and Mater-Bi^®^ NF 866 with gum rosin (**d**,**d’**).

Figure 3 shows FE-SEM images acquired for each formulation (MB, MB-LF, MB-UT, and MB-GR). The images at higher magnifications were colored to contrast the different phases observed. In the case of MB in the delivery state (Figure 3a,a’), a dispersed phase of spherical particles (1) between 1.4 and 4.6 µm of diameter corresponding to the semi-crystalline phase of the thermoplastic starch (label as TPSc) is observed. This phase is composed of the non-plasticized starch granules [20,37,38,39]. The matrix phase (2), has a rough appearance and contains smooth gaps (3), which correspond to the amorphous phase of the thermoplastic starch, TPSa, and the PBAT phase, respectively [20,40,41,42]. Inside the PBAT phase, it is possible to see regions where very fine particles are revealed, shown as short point in this phase (see the arrow label as PCL in Figure 3a’). These particles could correspond to the additives of the MB matrix (including PCL) and that have been retained within the PBAT phase, whose purpose is precisely to improve the compatibility between PBAT and TPS [5,8]. In addition, this hypothesis coincides with the fact that PCL has a strong interaction with PBAT matrix [43]. Moreover, it is compatible with the TPS phase [44]. The possible heterogeneous concentration of PCL in PBAT matrix and its possible consequences will be studied later with AFM technique.

The SEM analysis reveals cracks that delimit the border of each phase. In Figure 3a’ it is possible to verify this type of failure that indicates low miscibility among the components, generating a microstructure with little cohesion between the phases. The miscibility failure for MB formulation occurs at the TPSc/TPSa interphase (marked as f1), between TPSc/PBAT (marked f2), and between TPSa/PBAT (marked as f3). The greatest incompatibility is revealed between the PBAT and the TPSa, showing large cracks between both phases. Thus, the miscibility failure between the phases for this formulation is confirmed. The lack of cohesion of the microstructure observed, is a route of low mechanical properties, as observed in Figure 1, justifying the study of new formulations that lead to improving the compatibility between phases.

It is important to point out that the observed cracks were probably caused/revealed by the drag of the ultramicrotome blade. Precisely, this way of preparing the samples has been successful to reveal both the microstructure and the type of failure. To the best of our knowledge, Mater-Bi^®^ NF 866 had never before observed with this clarity and level of detail. In addition, because of the method of preparing samples, it is also observed that some TPSc particles have jumped from the matrix phase (TPSa) leaving the corresponding gap, as can be observed in the low magnification image (Figure 3a).

Moreover, Figure 3b,b’ show the microstructure of the MB-LF formulation. The microstructure obtained is very similar to that of MB supplied, clearly revealing the phases described above and the PBAT/TPSa miscibility failure, already discussed. However, there is a slight improvement in miscibility between the TPSc and TPSa phase due to the gum rosin derivate presence in this formulation (LF). This effect is confirmed not only by the absence of TPSc particles detached from the matrix phase but also because the TPSa phase shows good cohesion/wettability with the semi-crystalline phase TPSc. These results lead to consider that LF increases the miscibility between the semi-crystalline and the amorphous phases of thermoplastic starch (TPS), while the compatibility of TPS with the PBAT is not improved in this case. As a consequence, MB and MB-LF possess similar mechanical properties, as shown in Figure 1.

In contrast, the microstructure of the MB-UT formulation (Figure 3c,c’) shows an improvement in the miscibility of all the phases of the MB matrix material. In this case, there is no cohesion failure at the phase edges. In addition, the PBAT phase is much diluted in the TPSa, being difficult to define its grain border. Furthermore, the PBAT phase increases its compatibility towards the TPSc phase, finding some PBAT at the interface between the TPSc particles and the TPSa matrix phase (see the arrows in Figure 3c’). Another effect of good miscibility is the type of deformation mechanism observed around the TPSc particles. While, as previously analyzed, the TPSc was pulled out from the TPSa matrix phase in the MB; in formulations with 15 wt % of UT (MB-UT), good TPSc/PBAT cohesion leads to a rotation of the particles without failure of the interphase. This effect is observed by the bulging of the adjoining material and the inclination of the cut plane of the TPSc with respect to the other surfaces (see arrows in Figure 3c’).

Finally, the cutting surface of the MB-GR formulation (Figure 3d,d’) shows much-plasticized dunes and dragging mechanisms. This effect can be explained by the sticky features of the GR additive, which generates adhesive and drag mechanisms caused by the ultramicrotome blade, also favored by the lower stiffness expected for this formulation. This behavior is consistent with the plasticizing and compatibilizing effect of the gum rosin (GR) with this type of material, as discussed in Section 3.1 and shown in Figure 1. The microstructure can be seen as if it had reached a liquid state by the drop form of the PBAT phase, which now seems to be concentrated in the interface between the TPSc. Moreover, it is difficult to determine by this technique whether the PBAT is also diluted in the TPSa phase. This observation fits with the effect of decrease in processing temperature of formulations containing GR, already observed in previous work [8].

Being extruded at a higher temperature than necessary, the processing causes greater wettability of the PBAT phase in the material. No cracks that could indicate failures due to incompatibility between phases are revealed. On the other hand, small particles, colored in red in Figure 3d,d’, are found. They can be associated with the saturation of GR in the formulation. These small particles caused grooves on the TPSa during the cut with the ultramicrotome blade, indicating greater hardness compared to the other phases of the MB-GR formulation. To corroborate these hypotheses, a microstructural study by means of AFM was carried out to determine the nanomechanical properties of the individual phases as well as the adhesive component of each phase.

### 3.4. Atomic Force Microscopy with Nanomechanical Assessment (AFM-QNM) Microstructural Characterization

**Figure 4:**
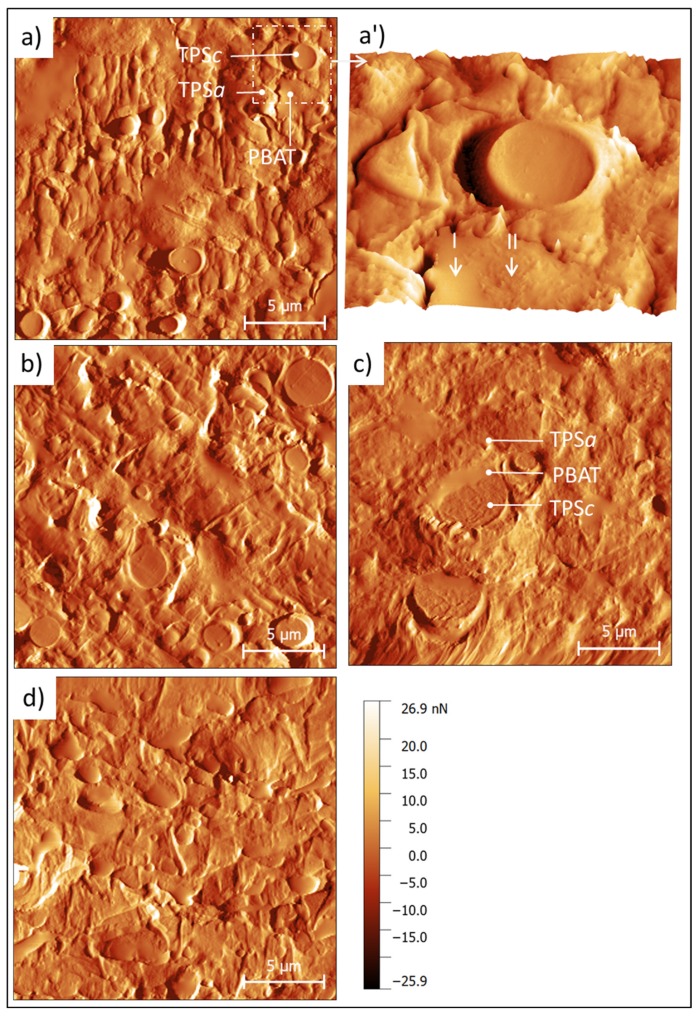
Peak Force-error channel assessed by atomic force microscopy (AFM) of Mater-Bi^®^ NF 866 with (I) PBAT free of PCL and (II) PCL particles in PBAT phase (**a**,**a’**); Mater-Bi^®^ NF 866 with Luerefor 125 resin (**b**), Mater-Bi^®^ NF 866 with Unik Tack P100 resin (**c**), and Mater-Bi^®^ NF 866 with gum rosin (**d**).

To study the mechanical properties of each phase individually and the interface, areas of 20 × 20 µm^2^ were scanned using AFM-QNM. Figure 4 shows the Peak Force-error channel obtained for each formulation. AFM results show that the MB (Figure 3a) reveals the same microstructure obtained by FESEM, corroborating the viability of the nanomechanical data discussed below.

The miscibility failure between phases is also revealed, as shown in the detail in Figure 4a’, which is represented in 3D. In addition, with this technique the heterogeneous localization of the PCL (and additives) in the PBAT is revealed with better resolution than the FESEM. That is, from one side the PBAT without PCL is observed in Figure 4a’ marked with the arrow (I), and from the other side the PBAT with solubilized PCL is also observed in the region marked with the arrow (II). In this last region, the PCL is revealed as very fine particles and concentrated in certain areas of the PBAT.

Furthermore, this study has discovered that there are miscibility differences between the PBAT and TPSa according to the location of the PCL within the same PBAT grain. Specifically, the area of the PBAT where PCL is not revealed, shows failure due to miscibility between PBAT and TPS (the edge with presence of crack in Figure 4a’, zone I), while areas of the PBAT rich in PCL particles coincide with good miscibility with the neighboring TPS (see the border of PBAT grain in Figure 4a’, zone II), corroborating the compatibilizing effect of the PCL.

Regarding the image for the MB-LF formulation (Figure 4b), the behavior of the material agrees with the performance observed by FESEM and apparently shows the same microstructure as MB, although the compatibility between phases increases slightly, especially for the PBAT with the TPSc. Besides, the MB-UT sample (Figure 4c) shows a smoother and more homogenized surface where the PBAT phase clearly shows good miscibility with the TPSc. The PBAT is revealed as a liquefied phase that wets the TPSc particles. Finally, the MB-GR formulation (Figure 4d) shows high miscibility between all the components with an important plasticized drag effect and an appearance of low viscosity, resulting in a textured microstructure due to the drag of the blade. This behavior indicates a set of mechanical properties very different from the rest of the formulations tested, as shown in Figure 1.

**Figure 5:**
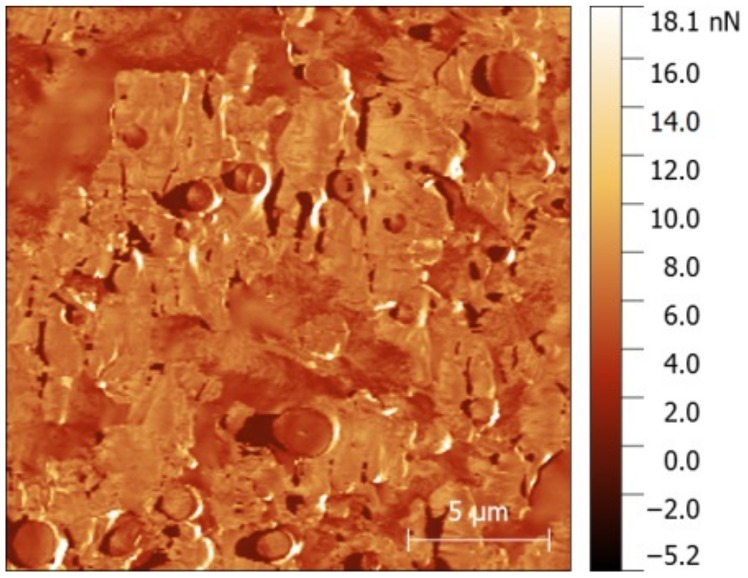
Force adhesion map acquired by atomic force microscopy with nanomechanical assessment (AFM-QNM) on supplied Mater-Bi^®^ NF 866.

In the same AFM test, the force-penetration curves were recorded at each point of contact between the tip and the surface of each sample. From the analysis of these curves, the adhesive component and the contact stiffness are obtained [33]. Figure 5 shows the adhesion map for the MB sample, as a representative formulation. Analyzing the adhesive response of each independent phase, it was determined that the PBAT and the TPSc particles have a similar adhesive component (~4.5 nN). On the other hand, the amorphous matrix TPSa has the highest adhesive behavior (~6.5 nN) of all of the components in the formulation. Comparing the adhesion strength of each sample, it was confirmed that all the formulations gave a similar average of 5.4 ± 0.9 nN, except for the MB-GR formulation which had an average value of 8.32 ± 2.5 nN. This observation agrees with the greater adhesive behavior attributed to this sample according to the microstructural observation, confirming the plasticizing effect of the GR on the physical-chemical properties of the material, already discussed. These data and their rheological consequences should be taken into account for the possible consequences that they may have in the manufacturing processes and final applications [8].

**Figure 6:**
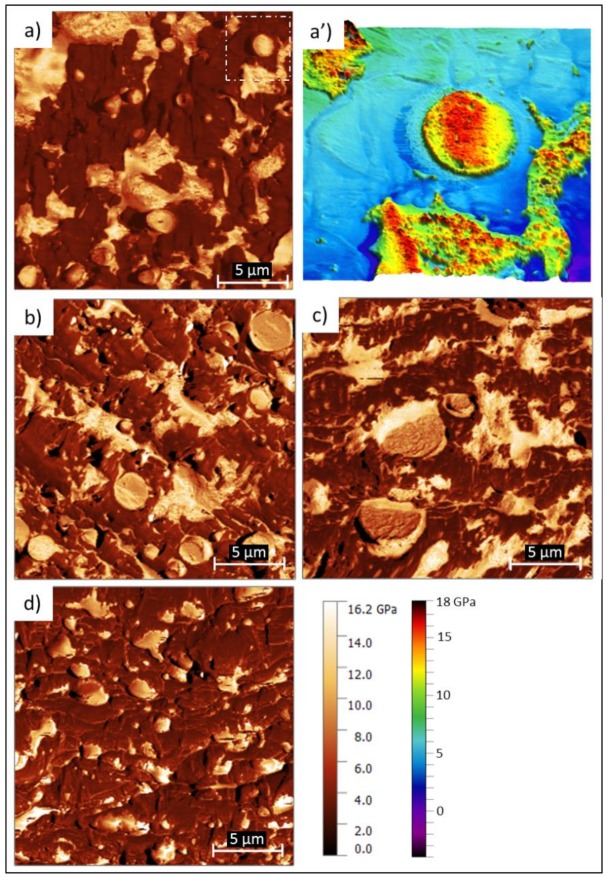
Elastic modulus map obtained by AFM-QNM of Mater-Bi^®^ NF 866 (**a**,**a’**); Mater-Bi^®^ NF 866 with Luerefor 125 resin (**b**), Mater-Bi^®^ NF 866 with Unik Tack P100 resin (**c**), and Mater-Bi^®^ NF 866 with gum rosin (**d**).

Additionally, the results of the elastic modulus of all the formulations are shown in Figure 5. From the analysis of the supplied MB (Figure 6a,a’) it was determined that the elastic modulus (E) of the TPSa phase (dark-colored matrix) was 2 GPa, while the TPSc particles showed an E value of 12 GPa. The PBAT phase showed the dispersion of E values between 12.5 and 17 GPa, resulting in the most rigid phase of all the components of the MB. The most rigid regions of the PBAT phase coincide with the areas where PCL particles are revealed. However, these particles are very thin and protrude from the surface, which can cause a poorly estimated contact area and therefore the E values may be overestimated. Future studies will be necessary to corroborate the stiffening effect of the PCL in the PBAT. From this analysis, it is understood that the TPSa matrix phase is about 30% less rigid than the PBAT and TPSc particles.

The E value of each phase remained similar for all formulations although the values in the interfaces were different, decreasing the module gradient when the gum rosin and derivatives were added, as can be seen in Figure 6. Therefore, it can be concluded that the dispersed phases (PBAT and TPSc) behave as reinforcement of the less rigid TPSa matrix phase. The modulus maps in Figure 6 also reveal with greater contrast the location of each phase (TPSa, TPSc, and PBAT). Moreover, Figure 6b corresponds to the MB-LF, which shows a PBAT phase very diluted in the TPSa, corroborating the improvement of the LF addition in the solubility of the components. However, the PBAT does not appear in the TPSc particle interface, as observed by FESEM. The MB-UT formulation (Figure 6c) shows that PBAT (which was previously only in the matrix phase) is also incorporated into the interface with the TPSc particles, confirming the hypothesis previously raised. Finally, when GR is used as an additive, in the MB-GR formulation, the E maps reveal and confirm how the PBAT is diluted and uprooted from the TPSa and moved to the surface of the TPSc particles. This effect may be due to the greater liquefaction achieved by the physical-chemical effect provided by the GR, which causes a decrease in temperature and viscosity, as previously mentioned. This displacement of PBAT component outside the matrix phase, due to GR presence, explains why it is the formulation with the greatest elongation at break obtained, under uniaxial stress tests (around 90% of elongation at break, while the MB formulations showed around 20%), reported in Figure 1. In other words, the 2 GPa matrix phase is released from the 12–18 GPa PBAT load that blocked the plastic deformation of the matrix phase. In addition, as the PBAT covers the TPSc particles, a higher level of energy absorption is expected as observed in Charpy’s impact resistance values (Figure 1).

### 3.5. Oxygen Permeability and Wettability

Since these materials are proposed as biodegradable films for agricultural and food packaging applications in which protection from humidity and oxidation processes are required, their oxygen barrier properties were studied by the determination of the oxygen transmission rate through the films (OTR.*e*). The hydrophilic/hydrophobic character of films was also evaluated, by water contact angle measurements; the results are summarized in Table 1.

Mater-Bi^®^ NF 866 presents low oxygen barrier properties which decreased with the incorporation of gum rosin due to its plasticizing effect which increases the free volume in the polymeric matrix, justifying the reduction in the resistance of plasticized films to oxygen transmission, as has been reported for other plasticized bioplastics [17,45,46,47,48]. The incorporation of pentaerythritol esters of gum rosin (LF and UT) produced two different effects. While LF further increased the oxygen permeability of MB polymeric matrix, UT mainly maintained the oxygen barrier properties of MB. As has already been commented, UT favors the solubility of the PBAT polymeric component in the TPSa leading to a homogeneous blend. Moreover, this result can be directly related with the higher crystallinity of these materials (χ_c MB-UT_ = 3.1, χ_c MB-GR_ = 2.8, and χ_c MB-LF_ = 1.5 and χ_c Mater-Bi® NF 866_ = 0.1 [8]). The increase in crystallinity as well as the difference of each additive in the crystallization phenomena could induce different tortuosity for the oxygen molecules path through the polymer film, counteracting the decrease in the resistance of this film to oxygen transmission caused by the introduction of a plasticizer [47]. The oxygen transmission through a film is highly influenced by the miscibility of each additive with the polymeric matrix. Thus, although all additives increased the crystallinity of Mater-Bi^®^ NF 866 (χ_c Mater-Bi® NF 866_ = 0.1 [8]), only UT was able to decrease or at least maintain the OTR.*e* value of MB due to the fact that it allows the best solubility of the PBAT component among all Mater-Bi^®^ NF 866 phases. It should be highlighted that the OTR.*e* values for these blends are clearly lower than that of commercial low density polyethylene (LDPE), 160 cm^3^ mm m^−2^ day^−1^, currently widely used in food packaging applications [45]. The decrease of permeability properties of starch/PBAT blends due to the improvement in their compatibility by the presence other additives has been already observed by Olivato et al. 2013 who used tartaric acid as compatibilizer [12].

The neat Mater-Bi^®^ NF 866 showed high WCA angles, and thus surfaces with hydrophobic characteristics in accordance with TPS matrices with high semicrystalline amylose component [10]. The addition of either gum rosin (GR) or UT derivative decreased the surface wettability of MB (Table 1). However, LF was not able to improve the hydrophobicity of MB; that can be related with the fact that TPSa phase shows good cohesion/wettability with the semi-crystalline phase TPSc, which favors the diffusion process of water as a consequence of the increased polymer chain mobility. Meanwhile, MB-UT showed the most hydrophilic surfaces. It has been observed that UT favors the solubility of the PBAT in the TPSa and allows that PBAT to appear at the interface of the TPSc particles and, thus, is less exposed to the surface leading to smoother surface. It should be mentioned that all formulations displayed typically hydrophobic surfaces for the intended use, since all formulations showed WCA higher than 65° and enough for the intended use as packaging or agricultural material [3,49].

## 4. Conclusions

The results of this study showed that the Mater-Bi^®^ NF866 (MB) has low miscibility among its main components: The amorphous phase of the thermoplastic starch (TPSa), the polybutylene adipate-co-terephthalate (PBAT) phase, and the semi-crystalline phase of the thermoplastic starch (TPSc). The low miscibility is revealed as a lack of cohesion between the phases and a dispersed microstructure. TPSa acts as a binder phase with an elastic module of 2 GPa, while the TPSc and PBAT are dispersed as fillers, with an elastic modulus of 12 and 12–18 GPa, respectively. In this way, by increasing the concentration of TPSc and PBAT in the formulation, a material with greater mechanical stiffness will be obtained, as long as a good cohesion with the TPSa matrix phase (which will have the greatest plastic deformability) is ensured. By using 15 wt % of Luerefor 125 resin (LF), the miscibility between the TPSa matrix phase and TPSc particles was improved. Nevertheless, the PBAT has no contact with TPSc and it is dispersed as well as immiscible within the TPSa. This immiscibility leads to an increase on the oxygen barrier performance. On the other hand, the same amount of Unik Tack P100 resin (UT) as additive favors the solubility of the PBAT in the TPSa and allows that PBAT to appear at the interface of the TPSc particles. In this formulation, the best dilution and miscibility of the PBAT is observed among all phases. In fact, the blend showed improved oxygen barrier, confirming once again the good miscibility among all the components. Finally, adding the gum rosin (GR) to the MB, a good solubility of the PBAT between the TPSa and TPSc is also achieved. Nevertheless, the PBAT sharply decreases its concentration within the TPSa, being located mainly on the surface of the TPSc particles. This causes the matrix phase to be released from the stiffer PBAT, covering the TPSc particles that have a similar stiffness (12 GPa). This relocation of the PBAT of the binder phase, together with the change in physical-chemical properties of the material (phases liquefied at the same processing temperature and adhesive behavior), justify the elongations at break of the material and its higher toughness.

## Figures and Tables

**Table 1:** Oxygen permeability and wettability results.

Sample	OTR.*e* (cm^3^ mm m^−2^ day^−1^)	WCA
**Mater-Bi^®^ NF 866**	57.0 ± 3.4	94.7 ± 1.3
MB-LF	118.9 ± 8.4	93.6 ± 1.5
MB-UT	55.7 ± 2.4	78.7 ± 1.2
MB-GR	78.6 ± 1.2	83.3 ± 1.6
